# Comparative Analysis of the Quality of Life in Families with Children or Adolescents Having Congenital versus Acquired Neuropathology

**DOI:** 10.3390/children9050714

**Published:** 2022-05-12

**Authors:** Maria V. Morcov, Liliana Pădure, Cristian G. Morcov, Andrada Mirea, Marian Ghiță, Gelu Onose

**Affiliations:** 1 National Clinical Centre of Neurorehabilitation for Children “Dr. N. Robanescu”, 041408 Bucharest, Romania; averopsi@yahoo.com (M.V.M.); lilianapadure@gmail.com (L.P.); drcrislegion@yahoo.com (C.G.M.); 2 Faculty of Medicine, University of Medicine and Pharmacy “Carol Davila”, 020022 Bucharest, Romania; geluonose@gmail.com; 3 Faculty of Midwives and Nursing, University of Medicine and Pharmacy “Carol Davila”, 020022 Bucharest, Romania; 4 Faculty of Veterinary Medicine, University of Agricultural Sciences and Veterinary Medicine, 050097 Bucharest, Romania; marianvet@yahoo.com; 5 Neuromuscular Rehabilitation Clinic Division, Teaching Emergency Hospital “Bagdasar-Arseni”, 041915 Bucharest, Romania

**Keywords:** disability, quality of life, family, congenital neuropathology, acquired neuropathology

## Abstract

Aim: This research aims to determine whether the time of injury (congenital or acquired) affects the quality of life (QOL) in families having a child or adolescent with neurological impairment. Design: Comparative, cross-sectional study. Material and methods: To find out if there are differences in the quality of life domains between these two groups, 66 subjects (31 mothers of patients with congenital disorders and 35 mothers of patients with acquired disorders) completed the PedsQL-Family Impact Module (PedsQL-FIM), the World Health Organization Quality of Life Instrument—Short Form (WHOQOL-BRIEF), and the Cognitive Emotion Regulation Questionnaire (CERQ). Results: Analyzing the PedsQL-FIM dimensions, we found significant differences between groups in terms of emotional functioning, communication, and worry, which favor the congenital group. There are no statistically significant differences between social functioning, cognitive functioning, and daily activities groups. No significant differences between groups when evaluating the WHOQOL-BRIEF’s domains (physical health, psychological health, social relationships, environment) have been found. According to CERQ results, adaptive strategies had higher mean scores in the congenital than in the acquired group. The mean score for maladaptive strategies in the congenital group is higher than that in the acquired one, except for catastrophizing, which is higher for acquired. Conclusion: Our findings show that the mothers of patients with acquired neuropathology have a lower quality of life in the emotional functioning, communication, and worry domains.

## 1. Introduction

Given the complexity of the concept of disability, there have been numerous attempts to define it. Thus, disability is a “shorthand expression” [1] or, according to the World Health Organization (WHO)’s International Classification of Functioning, Disability and Health (ICF), an “umbrella term for impairments, activity limitations or participation restrictions” [2,3] that are present from birth or develop over time. The impairments, mainly motor, sensory or cognitive, limit a child’s or an adolescent’s ability to perform necessary/required activities and interact with the surrounding environment [4].

For example, cerebral palsy is a “congenital” condition when the timing of the lesion is unknown (develops before birth, at birth, or shortly after delivery) [5,6]. It is usually diagnosed in the first few years of life and is the most prevalent cause of disability in children [7]. In contrast, acquired brain injury (ABI), which occurs when an external situation impairs the brain’s normal functioning, is also a leading cause of acquired disability [8,9].

The Romanian National Institute of Statistics reported an incidence rate of nervous system diseases of 1.68% of inhabitants in the age range of 0–19 years in 2020 [10]. The same institute reported an incidence rate of all acquired brain injuries of 1.54% of inhabitants, 0.08% of which were for traumatic brain injuries in the age range of 0–19 years in 2020 [10].

The disability, whether congenital or acquired, could affect the child’s or adolescent’s mobility and availability to perform certain tasks and his/her ability to actively participate in school and social life, leaving him dependent on those around him (parents siblings, grandparents). As a result, the entire family’s mental, physical, economic, and social-relational well-being is affected [11,12,13], and hence, overall, their QOL, too.

Although the agreement is required to define it, the concept of QOL is still far from being agreed upon [14,15]. A definition is given by the World Health Organization (WHO) and refers at “an individual’s perception of their position in the life in the context of the culture in which they live and in relation to their goals, expectations, standards and concerns” [15,16]. The concept is used in many fields, including sociology, psychology, and medicine, with interest in it being motivated by the desire for a better life in all of them [16,17]. It is a multidimensional concept that, from a medical perspective, can lead to the analysis of the physical, and psychosocial impact of the disease and treatment and the evaluation of the patient’s needs for psychological and social support [17].

Studies on QOL in families having children with either congenital or acquired impairment were searched in the medical databases (PubMed, PMC, Scopus) between 2018 and 2022). We searched using the following keywords: quality of life, family, congenital neuropathology, and acquired neuropathology. No comparative research on the quality of life in families with children with congenital versus acquired neurological impairments was found.

Without exhausting the database search, the articles correlated the quality of life in families with children who have congenital or acquired neurological impairment to family impact [18], family functionality [19], parental stress [20], economic burden [21,22], physical and psychological functioning [23].

According to the authors’ findings, family happiness, social family life, and harmony were severely impacted in families with chronically ill children. Caregivers were financially burdened, had less personal time, and experienced significant mental stress, all of which harmed their health and decreased their QoL [22,23,24,25].

The effect on other members in a family having a child with a chronic disease is similar across many chronic diseases, the biggest impact being the presence of the disease, not its specific type [26]. According to studies found in the literature, a chronic disease in a child has a negative impact on the child’s physical, psychological, emotional, social, and academic functioning [27,28,29].

Furthermore, children and adolescents who have a chronic illness believe that it limits their daily activities and are concerned about their future health [30]. They and their family report a lower QOL than a family with healthy children [31].

The occurrence of a disabling event at a certain point in life, in a family having children with acquired disabilities, leads to a gap/break in the previous normal life. This could induce coping behaviors and different quality of life in families in which the child has a disability at birth. Our study aimed to objectify if there is any difference between quality of life in a family having children with congenital versus acquired neuropathology.

## 2. Materials and Methods

### 2.1. Participants

A total of 66 subjects, mothers of children or adolescents with neurological disorders admitted to the National Clinical Centre for Children Neurorehabilitation “Dr N. Robanescu”, had completed two widely acknowledged related evaluation questionnaires: PedsQL-Family Impact Module (PedsQL-FIM) [32] and World Health Organization Quality of Life Instrument, Short Form (WHOQL-BRIEF) [33], Romanian version. We also used the Cognitive Emotion Regulation Questionnaire (CERQ) [34] to determine the cognitive-emotional strategies our participants adopt when confronted with the challenge of the children’s illness. All items in each questionnaire were surveyed.

The design of our study did not include a specific questionnaire to address the relationship between the family and the child with disabilities.

Subjects were invited to participate in the study voluntarily, enrollment taking place after they signed an informed consent form outlining specific aspects of the study (research description, confidentiality of collected data, their right to withdraw with researchers’ notification, and data exclusion).

Subjects were asked to complete the Romanian paper version of the PedsQL-FIM, WHOQL-BRIEF, and CERQ questionnaires, which assessed their functionality in various dimensions/domains and conscious cognitive emotion regulation strategies. Basic demographic data were also gathered, including education, marital status, residency, age of their children, the time between the moment of the accident and data collection (for acquired neuropathology), and impairment severity. Medical records from the hospital were used to obtain information about the impairment severity. The classification by severity level was used to differentiate children with cerebral palsy into mild, moderate, and severe categories. To determine the severity of traumatic brain injury (TBI), the Glasgow Coma Scale was used (mild, moderate, or severe).

All the data were collected between December 2019 and January 2022. 

We divided the enrolled subjects into two groups: Group 1 consisted of 31 mothers of patients with the congenital disorder (cerebral palsy of prenatal and perinatal origin), and Group 2 of 35 mothers of patients with the acquired disorder (traumatic brain injury after road/car accidents).

### 2.2. Research Tools

The first author received permission to use the Romanian version of the scales for this research, PedsQL-FIM from MAPI Research Trust, and WHOQOL-BREF from the WHO. For CERQ, permission to use the questionnaire was obtained from Cognitrom, a Romanian research organization authorized to commercialize its version in the Romanian language.

#### 2.2.1. PedsQL-Family Impact Module (PedsQL-FIM)

The PedsQL-FIM is a tool created to assess the impact of having a child with health issues in the family [35]. According to the authors who developed it, its internal consistency reliabilities passed the acceptable minimal alpha coefficient standard of 0.70 for group comparisons [22].

This instrument contains 36 items divided into 8 dimensions, which are as follows: physical functioning (6 items), emotional functioning (5 items), social functioning (4 items), cognitive functioning (5 items), communication (3 items), worry (5 items), daily activities (3 items), and family relationships (5 items) [36]. We surveyed all the 36 items of the questionnaire. The mothers filled out the questionnaire based on how many problems they had in the previous month because of their child’s illness.

For each evaluated item, a 5-point Likert scale ranging from 0 (never a problem) to 4 (almost always a problem) is used [37].

As higher the result of each component, as higher the quality of life is (the scores are obtained after the transformation of the variables).

#### 2.2.2. World Health Organization Quality of Life Instrument, Short Form (WHOQL-BRIEF)

WHOQOL-BREF is a shorter version of the WHOQOL−100 that is used to assess QOL in healthy people and groups of patients with various pathologies [38,39,40].

Used in clinical studies or research, WHOQOL-BREF is regarded as a relevant and trustworthy measure for assessing the quality of life in the medical field [41]. Cronbach alpha values for each of the four domain scores ranged from 0.66 to 0.84 [42]. It has 26 questions, with Q1 and Q2 being two global items that inquire about “individual’s overall perception of quality of life” (Q1) and “individual’s overall perception of their health” (Q2) [33]. We surveyed all 26 items of the questionnaire. The WHOQOL-BREF has four domains: physical health (7 items), psychological health (6 items), social relationships (3items), and environment (8 items) [33]. Each item is graded on a 5-point Likert scale ranging from 1 to 5. Raw WHOQOL domain scores are converted to a 4–20 score based on standards. Domain scores are scaled in a synergist, positive direction; lower scores indicate lower QOL. The domain score is calculated using the mean score of items within each domain, then translated linearly to a 0–100 scale [33]. This questionnaire is usually ordered in the same manner as the quality of life, i.e., the higher values correspond to higher QOL.

#### 2.2.3. Cognitive Emotion Regulation Questionnaire (CERQ)

CERQ is a 36-item questionnaire that analyzes the conscious cognitive strategies of emotion regulation [34]. We surveyed all the 36 items of the CERQ tool. It has an internal consistency of 0.75 to 0.87, making it a valuable and reliable tool for identifying cognitive emotional regulation strategies [34]. The questionnaire is divided into nine subscales (each subscale contains 4 items) which include the following strategies: “self-blame,” “acceptance,” “rumination,” “positive refocusing,” “refocus on planning,” “positive reappraisal,” “putting into perspective,” “catastrophizing,” and “blaming others”/“other blame.” A person’s strategies could be “adaptive”/“adjusted” and “less adaptive”/“unadjusted” or “maladaptive” [43,44,45].“acceptance”, “positive refocusing,” “refocus on planning,” “positive reappraisal”, and “putting into perspective,” are all considered adaptive, while “self-blame”, “rumination”, “catastrophizing”, and “blaming others” are all maladaptive [45]. Cognitive emotion regulation strategies were also measured on a 5-point Likert scale ranging from 1 (almost never) to 5 (almost always).

### 2.3. Statistical Analysis

To process statistical data analysis, we used Excel 2019 (Microsoft Corporation, Redmond, WA, USA) and SPSS version 27 (IBM, Armonk, New York, NY, USA).

In case the Kolmogorov–Smirnov test on both groups accepted the normality of data. Hence, the acceptance of use of the t-test for Equality of Means use of the same independent groups was used to determine if the QOL is lower because of the injury (congenital or acquired). If not, the Mann–Whitney U test (for Equality of Medians) was used [46]. 

Computed *p*-values < 0.05 were considered to be statistically significant.

## 3. Results

### 3.1. Socio-Demographic Features of the Groups 

Sixty-six mothers of children or adolescents with congenital versus acquired neuropathology (Groups 1 and 2) completed PedsQL-FIM, WHOQOL-BREF, and CERQ questionnaires.

Children and adolescents in Group 1 ranged in age from 72 to 204 months (M = 101.77, SD = 28.63), while those in Group 2 ranged in age from 72 to 216 months (M = 154.57, SD = 45.56).

The time between injury and data collection for mothers of children with acquired neuropathology was between 12 and 24 months.

In terms of severity, 7 (22.58%) of children or adolescents in Group 1 had a severe impairment, 13 (41.94%) had a moderate impairment, and 11 (35.48%) had mild impairment. In Group 2, 12 (34.29%) children or adolescents had severe, 9 (25.71%) moderate, and 14 (40%) mild impairment.

Table 1 summarizes the characteristics of our research groups in terms of education level, marital status, and residence. 

Using the Mann–Whitney U test, we found that the mean rank for Group 1 is 34.18 and for Group 2 is 32.90; *p* = 0.686, which is higher than 0.05, indicating that our groups are not statistically significantly different in education level. In terms of marital status, Group 1 has a mean rank of 30.35, while Group 2 has a mean rank of 36.29; *p* = 0.084. Our groups are not statistically significantly different neither on this feature.

Group 1 has a mean rank of 33.53, whereas Group 2 has a mean rank of 33.47; *p* = 0.988. There are no statistically significant differences between groups based on the criterion of the place of residence.

### 3.2. Descriptive Statistics

We calculated the mean, median, and standard deviation for each group and each questionnaire. The results are presented in Table 2.

### 3.3. Data Analysis for PedsQL—FIM Dimensions

To determine whether there is a difference in the quality of life in a family having children with congenital versus acquired neuropathology, we examined the data for PedsQL- FIM dimensions. We used Kolmogorov–Smirnov test to verify the normality of data for both groups. The *p*-values = Sig. should be > 0.05 for accepting a normal distribution of data (in fact, the threshold of 0.2 for normality acceptance; the threshold of 0.05 for categorical rejection). 

Physical function (*p* = 0.187), emotional functioning (*p* = 0.073), social functioning (*p* = 0.119), cognitive functioning (*p* > 0.200), communication (*p* > 0.200), worry (*p* > 0.200), daily activities (*p* = 0.082), and family relationships (*p* > 0.200)—shown a possibly normal distribution of data in Group 1. 

We also found a possibly normal distribution of data for some variables in Group 2—physical functioning (*p* = 0.160), emotional functioning (*p* = 0.076), communication (*p* > 0.200), and worry (*p* > 0.200). 

The Kolmogorov–Smirnov test rejected the normality of data for the following variables in Group 2: social functioning (*p* = 0.034), cognitive functioning (*p* = 0.040), daily activities (*p* = 0.002), and family relationships (*p* = 0.011).

We used the t-test to determine whether there were any statistically significant differences between groups for the variables with a normal data distribution (physical functioning, emotional functioning, communication, and worry).

*p*-values less than 0.05 were achieved for emotional functioning (*p* = 0.034), communication (*p* = 0.042), and worry (*p* = 0.036) indicating that there are significant differences between the two groups, Group 2 obtaining lower values (Table 3).

For physical functioning, we cannot assert the same; we can only say that the number of cases treated is too small to draw (statistically) a conclusion (Table 3).

The Mann–Whitney U test (for Equality of Medians) was used to determine whether there are significant differences in social functioning, cognitive functioning, daily activities, and family relationships between groups (where the data were not normally distributed).

Social functioning (*p* = 0.489), cognitive functioning (*p* = 0.199), and daily activities (*p* = 0.103) all have *p*-values higher than 0.05, indicating that there are no statistically significant differences between the enrolled groups for these dimensions.

The result is not statistically significant for the family relationships (*p* = 0.057).

### 3.4. Data Analysis for WHOQOL-BREF Domain

We have also analyzed the data for each WHOQOL-BREF domain.

Kolmogorov–Smirnov test rejected the normality of the related data, except for the psychological health variable (in Group 1, *p* = 0.069 and Group 2, *p* > 0.200), where this should be used with precaution.

To determine if there are statistically significant differences between groups for the variable psychological health, we used a *t*-test. Notice both means are apparently “close” one to the other mean for Group 1 is 14.96 and for Group 2 is 14.40 (Table 2).

Thus, the following result (*p* = 0.191) is not surprising and suggests no difference between groups regarding psychological health (Table 4).

We used the Mann–Whitney U test (for Equality of Medians) for the variables: physical health, social relationships, and environment to see if there are statistically significant differences between groups on these variables.

The variables, physical health (*p* = 0.370) and environment (*p* = 0.479) have *p*-values greater than 0.05, indicating no statistically significant differences between the enrolled groups in these domains.

The difference is not statistically significant for social relationships (*p* = 0.058).

### 3.5. Data Analysis for CERQ Questionnaire

We calculated the mean score for each subscale of the cognitive strategies for regulating emotions that our groups used when faced with the challenge represented by their children’s illness (Table 2).

Furthermore, we calculated the frequency of the subjects with the highest scores for each strategy for each group (Table 5).

The Kolmogorov–Smirnov test revealed a normal distribution of data with *p* > 0.05 for: acceptance, rumination, and refocus on planning in both groups. The t-test indicated for: acceptance (*p* = 0.804), rumination (*p* = 0.155), refocus on planning (*p* = 0.332), no statistically significant differences between groups.

We used Mann–Whitney U test (for Equality of Medians) to see any significant differences between groups for the following variables that did not have a normal distribution of data: self-blame, positive refocusing, positive reappraisal, putting into perspective, catastrophizing, and other blame.

There are no statistically significant differences between groups for self-blame (*p* = 0.430), positive reappraisal (*p* = 0.075), catastrophizing (*p* = 0.679), other blame (*p* = 0.226). Regarding positive refocusing (*p* = 0.023) and putting into perspective (*p* = 0.031) were found statistically significant differences between groups in favor of Group 1.

## 4. Discussion

To our knowledge, only studies comparing the quality of life of families with disabled children to those with normal development [47,48,49] or studies separately analyzing the quality of life of parents of children with congenital (cerebral palsy) [50] or acquired brain injuries (traumatic brain injury) [51,52] were available in the literature.

We did not find any comparative research on the quality of life in families with children with congenital versus acquired neurological disorders.

We believe that the importance of this study comes from the fact that no one has done anything similar before; therefore, we set out to perform it.

We found that there were no major differences between the groups. However, we supposed that the occurrence of a disabling event at a certain point in life could lead to a gap in previous normal life. As a result, coping strategies and quality of life differ from mothers with children with congenital impairment. We appreciate that for the family, regardless of when the injury occurs, at birth or after a period of normal development, it is a shock and a permanent challenge in the struggle with child disabling.

We found no statistically significant differences between our groups in terms of education, marital status, and place of residence. However, we cannot appreciate how the socio-demographic characteristics influence the quality of life in our studied groups.

We believe that differences in quality of life between families having a child with chronic disease due to economic status should be considered. A higher economic status influences the family, which has easier access to higher quality and innovative treatment in more developed countries and more performant medical assistive devices that are rarely compensated by standard health social programs.

The US literature showed that socio-demographic factors were associated with reduced QoL in families with children with chronic disease [53,54]. Some European countries with advanced economies and public health care identified them as having a risk for lower HRQOL in childhood. However, there are also European countries with public health care in which some studies report a minimal link between socioeconomic factors and QOL [53].

More studies agree that higher parental education can improve caregivers’ knowledge by supporting better coping strategies for managing children’s health and ensuring a better quality of life for themselves and their children [54,55]. Marital status is also important because tasks related to a disabled child’s care and education are usually shared in married families, with the burden of care not falling solely on a single parent [55].

Because no comparative studies were found in the literature, based on our statistical analysis, we can estimate that regardless of the type of impairment, congenital or acquired, the domains of the quality of life, social functioning, cognitive, and daily activity were equally valued in both groups.

Given the significant parental burden caused by the physical and psychosocial difficulties of children with a chronic or acquired disease, we can appreciate that the family’s structure, social, emotional, and relational functioning are disrupted.

In families with a disabled child, research has shown that illness severity and functional disability have a higher impact and may raise the chance of psychosocial issues [18,56].

Because the result for the family relationships has no statistical significance, we ca not clearly assess which group may have a higher or lower QOL in this domain.

We cannot say which group may have a higher or lower QOL on social relationships because the result for this domain has no statistical significance.

Considering the length of time of care and severity of disease could explain why most QoL parameters are nearly comparable in the two studied groups.

In families with children who have suffered a severe traumatic brain injury, long-term studies show that family relationships and functioning worsen between 3 and 36 months after the injury [57]. Long-term dependence harms family functioning in families with children with cerebral palsy [58].

Additionally, when completing the three questionnaires, the results enabled us to draw some conclusions regarding coping strategies.

Analyzing the mean score of CERQ, we found that the mean score for all adaptive strategies in Group 1 is higher than the mean score for all adaptive strategies in Group 2 (Table 2). We also found that the mean score for maladaptive strategies in Group1 is higher than the mean score for maladaptive in Group 2, except for catastrophizing, where the mean score is higher for Group 2 (Table 2).

Putting into perspective (45.1%) for Group 1 and catastrophizing (48.5%) for Group 2 are the strategies with the highest score frequency on the CERQ questionnaire.

Given the positive refocusing and putting into perspective strategies found to be better in Group 1, we can appreciate that parents with children with a chronic illness changed their perspective on life. Our study has the following limitations. (1) Self-administration of questionnaires could be considered a limitation due to misunderstanding questions. As a result, causing inaccurate responses despite the constant availability of investigators to clarify any question. (2) Considering that the evaluation tools gather data from a specific period (last month for PedsQL, last 2 weeks for WHQOL-BREF), mothers’ responses are limited at this period. (3) Our study analyzed information collected only from caregivers admitted to the hospital. This could be an objective limitation of the study in the biparental families where only one parent completed the questionnaires.

Further research is needed with an increased number of participants and, consequently, higher statistical power for a deeper analysis.

## 5. Conclusions

According to our findings from the questionnaire analysis, the QOL of the mothers of patients with traumatic brain injury (acquired neuropathology) is lower than that of patients with cerebral palsy (congenital neuropathology) for emotional functioning and communication and worry domains.

At the same time, both groups chose adaptive strategies that psychologically support them in struggling with their children’s illness and continuing the rehabilitation treatment.

## Figures and Tables

**Table 1:** Features of the 2 groups (total = 66 participants).

Features	Group 1 (Congenital) (*n* = 31)	Group 2 (Acquired) (*n* = 35)
Education level		
University	5 (16.13%)	7 (20%)
High school or less	26 (83.87%)	28 (80%)
Marital Status		
Married	21 (67.74%)	30 (85.71%)
Unmarried	10 (32.26%)	5 (14.29%)
Place of residence		
Urban	15 (48.39%)	20 (57.14%)
Rural	16 (51.61%)	15 (42.86%)

**Table 2:** Descriptive statistics for each tool for both groups.

Evaluation Tool	Diagnostic	Group 1 (Congenital) (*n* = 31)			Group 2 (Acquired) (*n* = 35)		
	Item	Mean	Median	Std. Deviation	Mean	Median	Std. Deviation
PedsQL-FIM	Physical functioning	54.03	54.16	21.71	61.07	62.5	18.79
	Emotional functioning	49.83	50	25.24	60.42	65	21.32
	Social functioning	64.11	62.5	30.14	65.53	68.75	21.67
	Cognitive functioning	63.7	65	23.9	68.85	70	21.89
	Communication	52.41	50	31.63	65.47	66.66	27.79
	Worry	43.87	40	26.13	54.42	55	20.82
	Daily activities	43.27	50	26.65	53.57	41.67	25.9
	Family relationships	61.29	70	31.3	72.57	80	28.8
WHOQOL-BREF	Physical health	14.74	14	1.98	14.8	15	2.67
	Psychological health	14.96	15	2.12	14.4	15	2.99
	Social relationships	14.83	15	2.81	15.45	16	3.73
	Environment	14.74	15	2.44	14.77	15	2.52
	Adaptive strategies						
CERQ	Acceptance	13.51	14	3.94	13.21	13	4.44
	Positive refocusing	12.51	12	3.75	10.37	10	3.52
	Refocus on planning	15.16	16	4.05	14.25	14	3.46
	Positive reappraisal	14.45	16	4.23	12.6	12	3.88
	Putting into perspective	14.61	16	4.46	12.37	12	3.58
	Maladaptive strategies						
	Self-blame	8.41	8	3.54	8.31	7	4.91
	Rumination	12.7	12	4.07	11.22	11	4.25
	Catastrophizing	10.35	10	4.3	10.68	10	3.87
	Other blame	8.58	8	4.64	7.57	6	4.5

**Table 3:** Calculation of the *p*-value using the *t*-test for four PedsQL- FIM variables.

		Levene’s Test for Equality of Variances		*t*-Test for Equality of Means						
		F	Sig.	*t*	df	Sig. (1-Tailed)	Mean Difference	Std. Error Difference	95% Confidence Interval of the Difference	
									Lower	Upper
Physical functioning	Equal variances assumed	0.229	0.634	1.412	64	0.081	1.68	1.19	−0.70	4.08
	Equal variances not assumed			1.399	59.79	0.083	1.68	1.20	−0.72	4.10
Emotional functioning	Equal variances assumed	0.239	0.627	1.847	64	0.034	2.11	1.14	−0.17	4.40
	Equal variances not assumed			1.828	59.08	0.036	2.11	1.15	−0.20	4.43
Communication	Equal variances assumed	1.100	0.298	1.756	64	0.042	1.53	0.87	−0.21	3.28
	Equal variances not assumed			1.742	60.09	0.043	1.53	0.88	−0.22	3.30
Worry	Equal variances assumed	2.503	0.119	1.825	64	0.036	2.11	1.15	−0.20	4.42
	Equal variances not assumed			1.800	57.25	0.038	2.11	1.17	−0.23	4.46

**Table 4:** Calculation of the *p*-value using the *t*-test for psychological health.

		Levene’s Test for Equality of Variances		*t*-Test for Equality of Means						
		F	Sig.	*t*	df	Sig. (1-Tailed)	Mean Difference	Std. Error Difference	95% Confidence Interval of the Difference	
									Lower	Upper
Psychological health	Equal variances assumed	1.945	0.168	0.879	64	0.191	0.56	0.64	−0.72	1.85
	Equal variances not assumed			0.897	61.19	0.186	0.56	0.63	−0.69	1.83

**Table 5:** Highest score frequency for each CERQ strategy.

**CERQ**	**Strategy**	Frequency * Group 1	Frequency * Group 2
	Self-blame	3 (3.2%)	7 (20%)
	Acceptance	11 (35.4%)	13 (37.1%)
	Rumination	7 (22.5%)	8 (22.8%)
	Positive refocusing	10 (32.2%)	5 (14.2%)
	Refocus on planning	9 (29.0%)	7 (20.0%)
	Positive reappraisal	8 (25.8%)	3 (8.57%)
	Putting into perspective	14 (45.1%)	6 (17.1%)
	Catastrophizing	13 (41.9%)	17 (48.5%)
	Other blame	10 (32.2%)	8 (22.8%)

* represents the number and percentage of the highest score (not a simplistic summation).

## Data Availability

The data sets used and analyzed during the current study are available from the corresponding author upon reasonable request.
